# Improving Care Through Conversation: Resident–Senior Forums in a Mental Health Trust

**DOI:** 10.1192/bjo.2026.11292

**Published:** 2026-06-30

**Authors:** Emily Pettifor

**Affiliations:** Kent and Medway Mental Health NHS Trust, Dartford, United Kingdom

## Abstract

**Aims:**

Kent and Medway Mental Health Trust facilitates regular Resident–Senior Forums for doctors of all grades, a structured space to raise work-related concerns and share feedback. Forums are attended by representatives from medical education, staffing and the Guardian of Safe Working Hours. They were initially piloted across two localities in 2023 as part of a QIP to improve on-call communication, receiving positive feedback and average attendance of 14 people. The forums have since been expanded trust-wide since 2024. After the initial phase chaired by locality tutors, I have chaired the forums as a resident doctor since August 2024 to provide continuity and peer-led perspective.

**Methods:**

The forums have supported discussion on a range of clinical and operational issues, including updates on standard operating procedures, consultant ward cover, rota andpay concerns, resident safety, ward access and educational needs. Issues have been escalated to senior colleagues across medical and non-medical departments, with actions fed back at later meetings. Topics such as physical health pathways and clinical handovers have led to QIP projects, with results shared with attendees. Non-medical teams have also joined forums to gather interactive feedback from doctors on issues such as clinical systems.

**Results:**

Since August 2024, attendance has risen to an average of 31 participants (range 18–43). In June 2025, feedback was re-collected from 14 medical staff from foundation to consultant grades: 71% had attended and 93% were aware of forums. Among attendees, 80% still found forums useful (sustained from 75–80% in the initial trial), 70% felt listened to (from 80%) and 90% felt able to raise concerns. Reported benefits included access to decision makers, a safe environment to voice concerns and improved understanding of organisational processes. Qualitative comments described the forums as an “opportunity to raise concerns and learn about colleagues’ issues”, “a voice for residents and consultants”, and “a shared space for juniors to be heard by seniors”. The main barrier to attendance was difficulty being released from clinical duties. Suggested improvements included increasing resident engagement, widening the agenda, clearer action tracking and adjusting timing. In response, the forums have been added to induction materials, an explanatory communication circulated trust-wide and the timing moved to lunchtime for 2026.

**Conclusion:**

The forums provide an effective route for raising concerns with seniors and have supported meaningful service improvements. Chairing as a resident doctor has also offered leadership development, insight into policy implementation and greater understanding of multidisciplinary roles.

